# Influence of Urbanization on Demography of Little Brown Bats (*Myotis lucifugus*) in the Prairies of North America

**DOI:** 10.1371/journal.pone.0020483

**Published:** 2011-05-25

**Authors:** Joanna L. Coleman, Robert M. R. Barclay

**Affiliations:** Department of Biological Sciences, University of Calgary, Calgary, Alberta, Canada; University of Utah, United States of America

## Abstract

**Background:**

We address three key gaps in research on urban wildlife ecology: insufficient attention to (1) grassland biomes, (2) individual- and population-level effects, and (3) vertebrates other than birds. We hypothesized that urbanization in the North American Prairies, by increasing habitat complexity (via the proliferation of vertical structures such as trees and buildings), thereby enhancing the availability of day-roosts, tree cover, and insects, would benefit synanthropic bats, resulting in increased fitness among urban individuals.

**Methodology/Principal Findings:**

Over three years, we captured more than 1,600 little brown bats (*Myotis lucifugus*) in urban and non-urban riparian sites in and around Calgary, Alberta, Canada. This species dominated bat assemblages throughout our study area, but nowhere more so than in the city. Our data did not support most of our specific predictions. Increased numbers of urban bats did not reflect urbanization-related benefits such as enhanced body condition, reproductive rates, or successful production of juveniles. Instead, bats did best in the transition zone situated between strictly urban and rural areas.

**Conclusions/Significance:**

We reject our hypothesis and explore various explanations. One possibility is that urban and rural *M. lucifugus* exhibit increased use of anthropogenic roosts, as opposed to natural ones, leading to larger maternity colonies and higher population densities and, in turn, increased competition for insect prey. Other possibilities include increased stress, disease transmission and/or impacts of noise on urban bats. Whatever the proximate cause, the combination of greater bat population density with decreased body condition and production of juveniles indicates that Calgary does not represent a population source for Prairie bats. We studied a highly synanthropic species in a system where it could reasonably be expected to respond positively to urbanization, but failed to observe any apparent benefits at the individual level, leading us to propose that urban development may be universally detrimental to bats.

## Introduction

Urbanization alters natural landscapes more dramatically than any other human agent of habitat change [1], so its effects on wildlife are greater than expected [2] given how little (<1%) of the Earth's total land area is occupied by urban areas [3]. Indeed, urbanization is a major cause of species' endangerments [4]. In 2007, at 3.3 billion, the world's urban population had already increased tenfold over a century before, and it is predicted to nearly double by 2050, when 70% of people will live in cities [5]. Given such rapid urbanization and the extreme habitat destruction with which the process is associated, the need to study species in urban ecosystems and develop conservation plans for those that need them is urgent.

Much progress has been made in the field of urban wildlife ecology [6], but it retains three key biases. The first one is geographic: most researchers have worked in forested regions (namely temperate forests), directing less attention to other biomes, including grasslands [see also 7,8]. The lack of urban ecology studies in grasslands is a knowledge gap in general, but especially in North America, where habitat destruction is more severe [9] and urbanization happens faster [10] in the Great Plains than in other biomes. The bias is problematic for another reason. Because urban tree-cover is fairly constant (≈30%) in all cities [11], urbanization in treed ecoregions implies the opposite process (deforestation) to that in a comparatively treeless landscape, such as grasslands [12]. Given how important vegetation is to wildlife, landscape context could modulate the effects of urbanization [13], but this cannot be verified without studies in a variety of biomes.

The second bias is methodological. The dominant perspective in urban wildlife ecology has been synecological [6], with researchers asking questions about abundance and diversity differences between urban and non-urban wildlife communities. Although this approach may reveal the changes in community size and structure that result from urbanization, only autecological studies can expose the mechanisms underlying those effects, such as impacts on fitness [7], [14].

The third bias is taxonomic, with most research devoted to birds [6], [15] and less to other vertebrates, including mammals. There is even bias in urban mammal studies: most have focused on charismatic, conspicuous species rather than on less charismatic or secretive mammals, such as bats [13], [15]. Studying the urban ecology of bats is worthwhile, and not just because of their ecological importance as predators of nocturnal insects and as pollinators [16]. For their size, bats have the slowest life histories of all mammals [17]. Long-lived, monoestrous bats, i.e., temperate-zone vespertilionids, are ideal for urban autecological studies because when environmental conditions are not conducive to successful breeding, they may forego reproduction in favour of somatic maintenance [18]. Thus, habitat change can profoundly affect their populations [19], making bats good indicators of habitat quality in general and responsive to urbanization [20]. Also, temperate-zone bats use daily torpor, which saves energy but has reproductive costs [21], [22]. They are therefore well-suited to addressing questions about how facultative heterotherms are influenced by the urban heat island effect, the tendency for cities to be warmer than outlying areas [23].

To address these research gaps, we studied the impacts of urbanization on bats of the Canadian Prairies. We hypothesized that urbanization increases their fitness in several ways. First, the availability of the vertical landscape elements that bats use as roosts [24] should be enhanced by the urban proliferation of trees and buildings in a relatively treeless Prairie landscape. Increased structural habitat complexity in the city should also increase habitat connectivity, which is important to many insectivorous bats [25]. Many bats avoid open, agricultural areas, and instead frequent treed, riparian areas and other linear habitat features, where insects may be more abundant [26] and where flight costs [27] and predation risk [28] may be lower. The urban heat island may also be relevant. At higher ambient temperatures, insects are more abundant, and so is bat foraging activity, especially at northern latitudes [29]. Lower temperatures and reduced energy intake, leading to increased torpor use, decrease allocation to reproduction [18] and delay parturition, weaning [30] and spermatogenesis [22]. Finally, compared to conspecifics in natural roosts, Prairie bats in buildings may benefit not only from increased roost temperature, but also from reduced predation [21], and building roosts should be more abundant in the city.

The focal species for our study was the little brown bat *Myotis lucifugus* because it is the dominant species throughout our study area, but nowhere more so than in the urban assemblage [31]. Gehrt and Chelsvig [13] suggested that such a scenario, especially in an agricultural landscape, could indicate that the city is a population source. Thus, we sought evidence that recruitment outweighs mortality. We tested the predictions that compared to non-urban conspecifics, urban little brown bats (1) are in better body condition, (2) are more likely to reproduce, (3) reproduce more successfully, and (4) have accelerated reproductive phenology.

## Materials and Methods

Our study area was in the South Saskatchewan River basin (SSRB), and our focal city was Calgary, Alberta (city centre 51°02′45"N, 114°03′27"W). The study area and level of urban development are described elsewhere [31].

### Study sites

We divided our study area into three zones, each with replicate sites≥1 km apart. Our 11 urban sites were within city limits and totally surrounded by urban development, and our six rural sites were≥40 km away from city limits. Our 10 transition-zone sites were either in the city but bordering city limits, or outside but <40 km from city limits. Mean distances from each site to the nearest human residence that occurred within a populated place (i.e., not an isolated farmhouse) were: 12.45±2.91 km in the rural zone, 4.30±1.72 km in the transition zone, and 0.20±0.04 km in the urban zone.

As we were interested in the effects of urbanization and not those of habitat, we selected field sites according to two search criteria of importance to Prairie bats in Alberta [26]. All sites (1) were riparian, located near rivers and tributaries in the SSRB, and (2) had mature, native trees that could potentially be used as roosts. Sites were located in municipal and provincial parks, provincial natural recreation areas, on municipal and private property and in one national historic site. Although transition-zone sites were located in all cardinal directions relative to Calgary, there were no rural sites to the west because <40 km west of city limits, the Boreal Plains and Montane Cordillera (Eastern Foothills) ecozones meet the Prairies [32].

### Study species

Little brown bats range over much of North America [33]. They are year-round residents in southern Alberta, and hibernate locally [34]. As in most temperate-zone vespertilionid bats, the sexes gather at hibernacula, where they mate and overwinter, but are segregated the rest of the year [33]. In the summer, reproductive females form colonies in maternity roosts [33], which in our study area include artificial (bridges, buildings) and natural structures, namely trees [31], and possibly rock crevices [35]. Non-reproductive females and males are less social, but may roost in any of the above structures. A reproductive female gives birth to a single pup [36], which fledges in about three weeks and is weaned shortly thereafter [33], and lactation is the most energetically expensive aspect of bat reproduction [37]. Summer is also when males undergo spermatogenesis, a costly process that is timed to the period of maximum food availability, with mature sperm stored until mating begins [38]. By early fall, little brown bats must accumulate enough fat to hibernate successfully, and the greater overwinter mortality of juveniles relative to adults [39] is related to their reduced ability to do so [33].

### Field methods

We assessed little brown bat demography in all three zones in 2007 and 2008, and in the urban and transition zones only in 2006.

From late May to late August (2006) and to mid-September (2007–2008), weather permitting, we captured bats in mist nets (Avinet, Dryden, NY, USA), in one site per night, alternating nights among zones, six nights a week.

We extracted bats from nets as soon as possible after capture and kept them in cloth bags for at least an hour, long enough for them to void their digestive system [40] and provide accurate mass measurements. We weighed bats to the nearest 0.1 g on a calibrated digital balance, and measured their forearm length to the nearest 0.1 mm with calipers. We identified juveniles by the presence of a cartilaginous epiphyseal gap [41]. We fitted each bat with a plastic split-ring arm band with a unique colour-number combination so we could identify recaptures.

We identified individuals to sex and adult reproductive status. We classified females that were pregnant, lactating or post-lactating [42] as reproductive, and those that were neither pregnant nor lactating by the date on which we first observed evidence of parturition each year as non-reproductive. We classified males as reproductive if they exhibited testicular swelling, which is indicative of spermatogenesis, and/or distension of the caudae epidydimes, where mature sperm are stored [42]. In 2007 and 2008, we also measured the linear extent of testicular/caudal swelling along the cranio-caudal axis to estimate the relative extent of spermatogenesis.

This study was carried out in accordance with the guidelines established by the Canadian Council on Animal Care and was approved by the University of Calgary Life Sciences Animal Care Committee (permit # BIO9R-01).

To account for the potential effects of weather on bat reproduction and development, we obtained climate data from Environment Canada's National Climate Data and Information Archive (http://www.climate.weatheroffice.ec.gc.ca/Welcome_e.html). These data were collected each year (2006–2008) from 01-May to 15-September (i.e., the period during which little brown bats are present in our study area) at two weather stations: one in the urban zone and the other in the rural zone (there were no adequate datasets for any transition-zone weather stations). Our analysis of those data revealed evidence of an urban heat island: it was warmer and more precipitation fell in the city than outside. We also found variation in temperature at both stations among years (it was warmest in 2006 and coolest in 2008), but whereas 2008 tended to be the wettest year at both stations, yearly variation in precipitation was only significant in the city of Calgary [31].

### Statistical analysis

For most dependent variables, we performed two separate analyses: model 1 considered bats captured in 2007 and 2008 in all three zones, and model 2 considered bats captured in 2006–2008 in the urban and transition zones. We used JMP 7.0 (SAS Institute Inc., Cary, NC, USA) and SPSS 16.0 (SPSS, Inc., Chicago, IL, USA) to perform statistical tests, all of which were two-tailed, with a rejection criterion of 0.05.

#### Body condition

We estimated adult male body condition, following Entwistle et al. [22], with a linear regression of mass against forearm length for all adult male bats. We used the studentized residual difference of each bat's mass from the value expected from the overall regression (r^2^_adj_ = 0.05, F_1_ = 14.34, *P* = 0.0002, N = 244) as a body condition index. We applied the same procedure to juveniles (r^2^_adj_ = 0.05, F_1_ = 14.94, *P* = 0.0001, N = 248). We estimated body condition of adult females differently. To account for the expected relationship between body size and mass (regressions of mass against forearm length were not significant), we followed [43] and divided each bat's mass by her forearm length and multiplied the result by the mean forearm length (38.5 mm; N = 978) of all adult females. We omitted within-year recaptures from body condition analyses.

We examined the effect of zone on body condition for each of four groups (adult males, juveniles, non-reproductive adult females, and lactating adult females) separately. We did not consider post-lactating females because of insufficent sample size. We performed model 1 and 2 general linear ANCOVAs, with body condition as the dependent variable, year and zone (and juvenile sex) as fixed factors, and Julian day as a covariate. We began this and other analyses with fully crossed models, and removed non-significant interactions sequentially, to obtain reduced models with no non-significant interactions [44]. For significant fixed effects, we conducted post-hoc t- or Tukey HSD tests, as appropriate.

#### Reproductive rates

For females, we considered only adults captured on or after the date on which the first lactating female was captured in any year, when all pregnancies should have been detectable. We performed two-way contingency table analyses, considering the association between reproductive status and zone, for each year separately, and the association between reproductive status and year, for each zone separately. We broke down significant associations into 2×2 tables to examine them in greater detail. We conducted similar analyses for adult males.

#### Spermatogenesis

We used a general linear ANCOVA to assess whether urbanization affects the extent of sperm production by reproductive males. The dependent variable was the linear extent of testicular/caudal swelling, hereafter referred to as swelling. Fixed factors were year and zone, and covariates were Julian day and body-condition index.

#### Reproductive success

We used the ratio of volant juveniles to post-parturient females in capture samples as an index of reproductive success. We performed two hierarchical three-way loglinear analyses (models 1 and 2) considering the association between zone, year and age (adult or juvenile). We decomposed significant higher-order interactions into two-way tables to examine them in detail.

#### Post-natal growth

Skeletal dimensions of bat pups vary with extrinsic factors, such as weather and food availability [45], and it is conceivable that habitat quality, i.e., urbanization, is also important. We compared forearm length of volant juveniles among zones and years in an ANOVA, with year, sex and zone as fixed factors.

## Results

We captured 1,627 *M. lucifugus*: 332 in 2006, 528 in 2007 and 767 in 2008 (Table S1). Of those, 34 were recaptures.

### Body condition

In general, females were heavier than males, and adults were heavier than juveniles, with pregnant bats heaviest of all (Table S2). Near-term females weighed ≤13.4 g, an increase of >60% over the mean mass of non-reproductive adult females.

**Figure 1:**
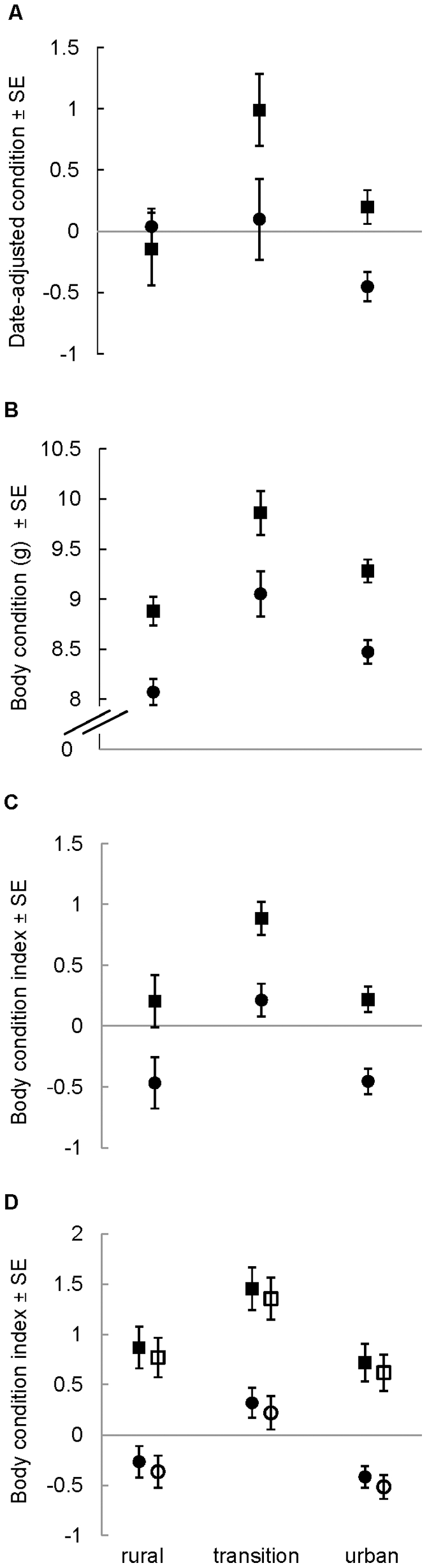
Variation in body condition of *M. lucifugus* among zones and between years. Panels present results for (A) non-reproductive female, (B) lactating female, (C) adult male and (D) juvenile bats captured near Calgary, Alberta, Canada. Values are least-squared means±standard error. Squares represent 2007 and circles represent 2008, with open symbols for male and closed symbols for female juveniles.

**Figure 2:**
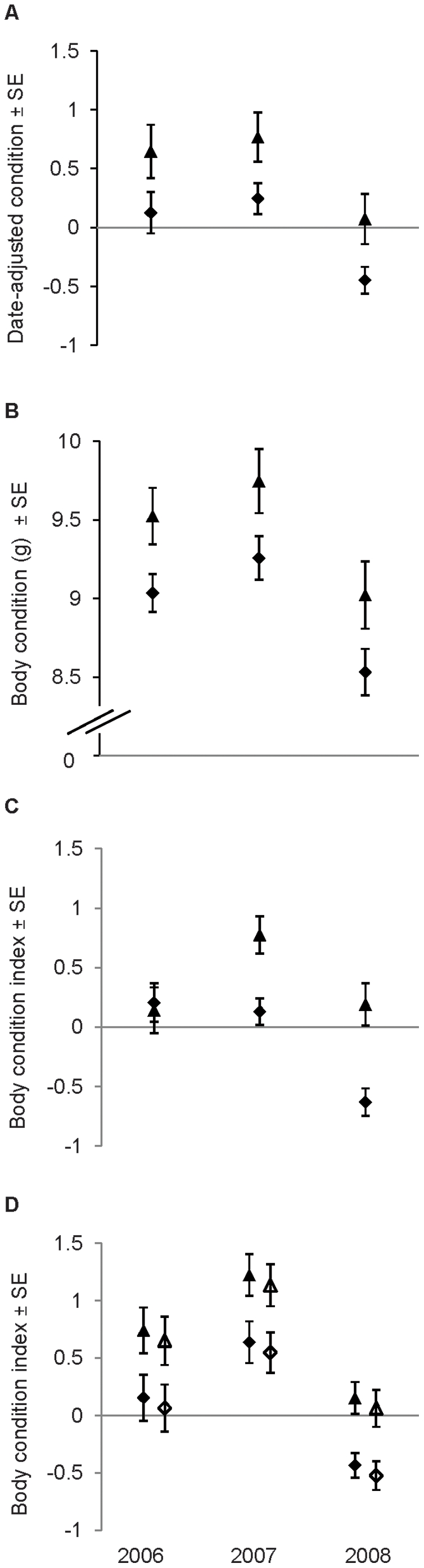
Variation in body condition of *M. lucifugus* between zones and among years. Panels present results for (A) non-reproductive female, (B) lactating female, (C) adult male and (D) juvenile bats captured near Calgary, Alberta, Canada.. Values are least-squared means±standard error. Triangles represent transition-zone bats, diamonds represent urban bats, with open symbols for male and closed symbols for female juveniles.

When we included date as a covariate in models 1 and 2 analysing non-reproductive female body condition, its effect was significant, but so were lack of fit tests, suggesting that a different model might be a better fit. Scatterplots revealed nothing to suggest a nonlinear relationship between date and body condition, and no data transformation significantly improved model fit. To retain the evidently important influence of date, we performed a linear regression of body condition (ln-transformed) against date (*R^2^*_adj_ = 0.06, F = 13.25, *P* = 0.0003, *n* = 209), and used the residuals as the dependent variable in two-factor ANOVAs examining the effects of year and zone. Hereafter, we refer to this variable as date-adjusted condition. Model 1 (*R^2^*_adj_ = 0.10, F = 7.21, *P*<0.0001, *n* = 177) revealed that bats were in better condition in 2007 than in 2008 (year: F_1,173_ = 11.16, *P* = 0.001), and in the transition zone than in the urban zone, with no difference between rural and transition-zone bats or between urban and rural bats (zone: F_2,173_ = 9.00, *P* = 0.007; Fig. 1A). According to model 2 (*R^2^*_adj_ = 0.14, F = 9.30, *P*<0.0001, *n* = 158), date-adjusted condition was worse in 2008 than in either of the other two years, between which it was not different (year: F_2,154_ = 9.30, *P* = 0.0002), and was worse in the urban than in the transition zone (F_1,154_ = 6.49, *P* = 0.01; Fig. 2A).

When we included date as a covariate in models of lactating female body condition, its effect was not significant, so we removed the term. Doing so also made biological sense: bats lose mass over the lactation period [37] but do not all give birth on the same day, so date should have an unpredictable effect on condition. Ultimately, the data were best explained by two-factor ANOVAs. Model 1 (*R^2^*_adj_ = 0.30, F = 19.21, *P*<0.0001, *n* = 129) revealed significant effects of year (F_1,125_ = 33.89, *P*<0.0001) and zone (F_2,125_ = 9.01, *P* = 0.0002), with condition better in 2007 than in 2008, and best in the transition zone, intermediate in the urban zone and worst in the rural zone (Fig. 1B). Model 2 (*R^2^*_adj_ = 0.11, F = 7.29, *P* = 0.0001, *n* = 149) revealed that body condition was poorest in 2008 (year F_2,145_ = 6.99, *P* = 0.001), and better in the transition zone than in the urban zone (F_1,145_ = 6.95, *P* = 0.009; Fig. 2B).

The reduced model 1 ANCOVA for adult males (*R^2^*_adj_ = 0.31, F = 18.89, *P*<0.0001, *n* = 196) retained an interaction between year and date (F_1,190_ = 4.01, *P* = 0.047). We used the JMP 7.0 profiler to estimate the regions of significance for each variable. The relationship between date and body condition (date: F_1,190_ = 73.77, *P*<0.0001) was significantly positive in both years, but stronger in 2007. Body condition was generally better in 2007 than in 2008 (year: F_1,190_ = 26.39, *P*<0.0001; Fig. 1C), significantly so in bats captured on or after approximately 09-July. Bats in the transition zone were in better condition than their rural or urban counterparts, which did not differ from each other (zone: F_2,190_ = 11.26, *P*<0.0001; Fig. 1C). The reduced model 2 (*R^2^*_adj_ = 0.30, F = 17.02, *P*<0.0001, *n* = 226) retained a year-by-zone interaction (F_2,219_ = 3.97, *P* = 0.02). In general, body condition was better in 2007 than in 2008 (year: F_2,219_ = 10.64, *P*<0.0001; Fig. 2C), but not different between any other two years, and the difference was only significant in the urban zone. The effect of zone (F_1,219_ = 13.28, *P* = 0.0003) was significant in 2007 and 2008, when transition-zone bats were in better condition than their urban counterparts (Fig. 2C). Body condition increased as a function of date (F_1,219_ = 84.65, *P*<0.0001).

The reduced model 1 ANCOVA for juveniles (*R^2^*_adj_ = 0.25, F = 11.08, *P*<0.0001, *n* = 218) revealed that body condition was better in 2007 than in 2008 (year: F_1,210_ = 39.17, *P*<0.0001) and in the transition zone than in either of the other two, between which it did not differ (zone: F_2,210_ = 11.20, *P*<0.0001; Fig. 1D). Females tended to be in slightly, but not significantly, better condition than males (F_1,210_ = 0.61, *P* = 0.44; Fig. 1D). Model 2 (*R^2^*_adj_ = 0.27, F = 11.01, *P*<0.0001, *n* = 194) retained a significant date-by-zone interaction (F_1,186_ = 4.73, *P = *0.03). The effect of date (F_1,186_ = 16.34, *P*<0.0001) was significantly positive in both zones, but was stronger in the transition zone, and transition-zone bats were generally in better condition than urban bats (zone: F_1,186_ = 18.21, *P*<0.0001; Fig. 2D), but not significantly so until about 18-July. The model also retained a date-by-sex interaction (F_1,186_ = 8.69, *P* = 0.004). Condition increased significantly with date in both sexes, but more steeply in males, and there was no effect of sex (F_1,186_ = 0.46, *P* = 0.50). Bats born in 2008 were in the worst condition, but condition did not differ between bats born in 2006 or 2007 (year: F_2,186_ = 16.18, *P*<0.0001; Fig. 2D).

### Reproductive rates

**Figure 3:**
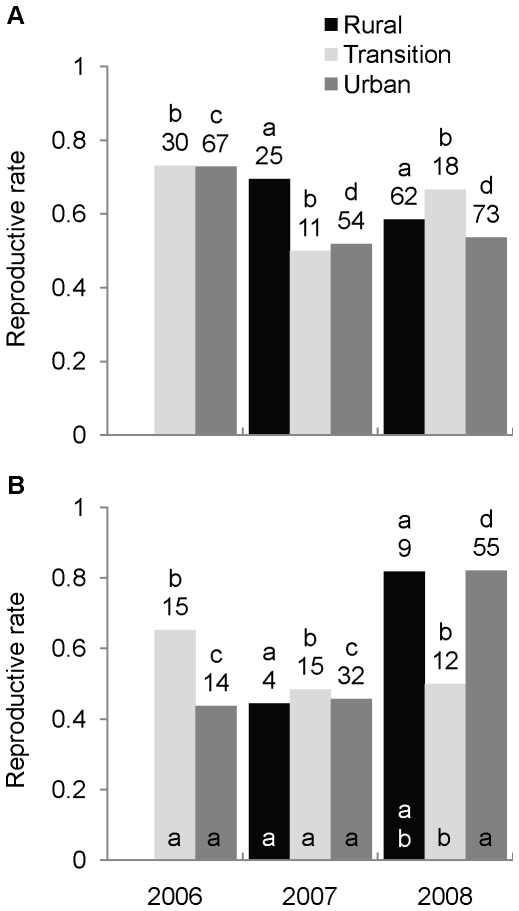
Comparison of reproductive rates among zones and years for *M. lucifugus*. Panels present results for (A) 429 adult female and (B) 267 adult male bats captured near Calgary, Alberta, Canada. Numbers above bars are counts of individuals, different letters above bars indicate yearly variation within a zone, and different letters at bases of bars in (B) indicate within-year variation among zones, which was not significant in (A).

Reproductive rates of adult females did not vary among zones (model 1: χ^2^_2_<3.65, *P*>0.16 in both years; model 2: χ^2^_1_<2.15, *P*>0.14 in all three years) or between 2007 and 2008, according to model 1 analyses (χ^2^_1_<1.85, *P*>0.17 in all three zones). Model 2 analyses revealed that female reproductive rates varied among years in the urban zone (χ^2^_2_ = 10.93, *P* = 0.004) but not in the transition zone (χ^2^_2_ = 3.58, *P* = 0.17). The urban rate was highest in 2006 (χ^2^_1_>8.40, *P*<0.005 in both comparisons), but similar between 2007 and 2008 (χ^2^_1_ = 0.07, *P = *0.79; Fig. 3A). Adult male reproductive rates differed among zones in 2008 (model 1: χ^2^_2_ = 9.93, *P = *0.007; model 2: χ^2^_1_ = 9.37, *P = *0.002), when they were higher in urban than in transition-zone bats (χ^2^_2_ = 9.37, *P = *0.002) but not different between any other two zones (Fisher's exact tests: χ^2^_1_<3.19, *P*>0.08 in both cases; Fig. 3B). There were no other significant associations between zone and male reproductive status (model 1 2007: χ^2^_2_ = 0.08, *P* = 0.97, model 2: χ^2^_1_<2.48, *P*>0.12 in both cases; Fig. 3B). Male reproductive rates were similar among years in the transition (χ^2^_2_ = 1.71, *P = *0.43) and rural zones (Fisher's exact test: χ^2^_1_ = 3.04, *P = *0.10), but different in the urban zone (χ^2^_2_ = 23.05, *P*<0.001), where the rate was highest in 2008 (χ^2^_1_>15.0, *P*<0.001 in both comparisons), but similar between 2006 and 2007 (χ^2^_1_ = 0.03, *P = *0.85; Fig. 3B). Finally, there was about twice as much yearly variation in the reproductive rates of urban males (Cramer's V = 0.37) as of urban females (Cramer's V = 0.18).

### Spermatogenesis

**Figure 4:**
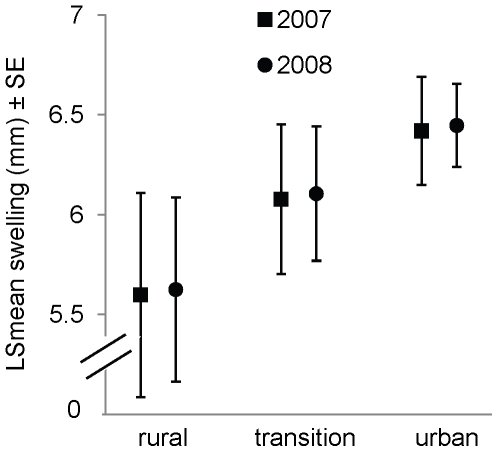
Comparison of sperm production among zones and between years for adult male *M. lucifugus*. We measured the linear extent of testicular/caudal swelling in 113 bats captured near Calgary, Alberta, Canada, and used it as an index of sperm production.

The reduced ANCOVA (R^2^_adj_ = 0.16, F = 5.32, *P*<0.0001, *n = *113) revealed that although mean swelling tended to be highest in urban bats, intermediate in transition-zone bats and lowest in rural bats, the zone effect was not significant (F_2,107_ = 1.71, *P = *0.19; Fig. 4). The effect of year was not significant either (F_1,107_ = 0.007, *P = *0.94), although swelling tended to be greater in 2008 than in 2007 (Fig. 4). With both fixed factors in the model, swelling varied with body condition (F_1,107_ = 14.53, *P*<0.001), but not date (F_1,107_ = 3.35, *P = *0.07). However, a multiple regression with date and body-condition index as predictors (r^2^_adj_ = 0.16, F = 11.55, *P*<0.0001, *n = *113) revealed an increase in swelling with date (F_1,110_ = 4.35, *P = *0.04) and body condition (F_1,110_ = 16.37, *P*<0.0001).

### Reproductive success

**Figure 5:**
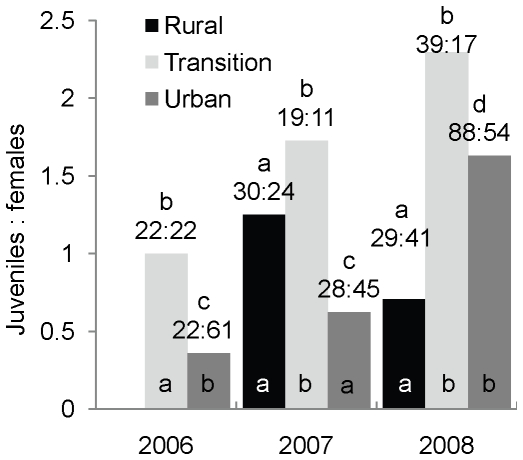
Comparison of an index of reproductive success of *M. lucifugus* among zones and years. We used ratios between post-parturient adult females and volant juvenile near Calgary, Alberta, Canada as indices of reproductive success. Numbers above bars are actual ratios, different letters above bars indicate yearly variation within a zone, and letters at bases of bars represent differences among zones within a year.

The model 1 loglinear analysis produced a final model that retained all effects, with a significant zone*year*age interaction (χ^2^_2_ = 10.78, *P = *0.005), but the model 2 analysis did not (χ^2^_2_ = 2.26, *P = *0.32). However, model 2 two-way interactions involving age were significant (year*age: χ^2^_2_ = 31.45, *P*<0.001; zone*age: χ^2^_2_ = 11.05, *P = *0.001). Two-way contingency table analyses for each year revealed significant variation in juvenile production among zones in 2008 (χ^2^_2_ = 11.94, *P = *0.003) and 2007 (χ^2^_2_ = 6.70, *P = *0.04). Reproductive success was lower in the urban than in the transition zone, and significantly so in 2006 and 2007 (2006: χ^2^_1_ = 7.01, *P = *0.008; 2007: χ^2^_1_ = 5.35, *P = *0.02; 2008: χ^2^_1_ = 1.03, df = 1, *P = *0.31; Fig. 5). It tended to be lower in the rural than in the transition zone, but only significantly so in 2008 (2007: χ^2^_1_ = 0.48, *P = *0.49; 2008: χ^2^_1_ = 9.97, *P = *0.002; Fig. 5). In 2007, reproductive success tended to be lower in the urban than in the rural zone, but not quite significantly so (χ^2^_1_ = 3.70, *P = *0.054; Fig. 5). In 2008, the difference between the urban and rural zones was significant and in the other direction (χ^2^_1_ = 8.0, *P = *0.005; Fig. 5). Reproductive success differed among years in the urban zone (χ^2^_1_ = 29.96, *P*<0.001), where it was highest in 2008 (χ^2^_1_>10.75, *P*<0.001 in both comparisons; Fig. 5), but not different between 2006 and 2007 (χ^2^_1_ = 2.50, *P = *0.14; Fig. 5). Reproductive success did not vary among years in the rural or transition zones (χ^2^_2_<4.10, *P*≥0.12 in both cases; Fig. 5).

### Post-natal growth

**Figure 6:**
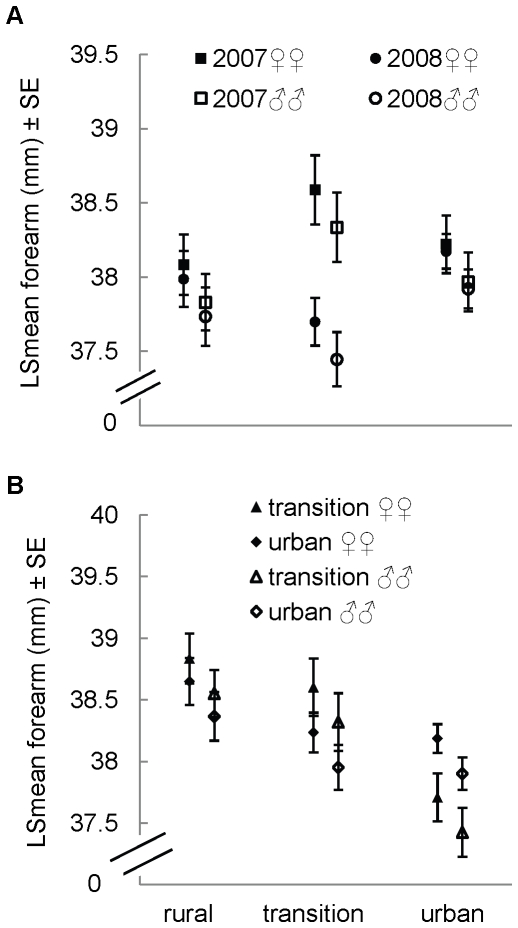
Comparison of skeletal size among zones and years for juvenile *M. lucifugus*. Panels present results for (A) 133 female and 98 male and (B) 124 female and 83 male juvenile *M. lucifugus*, near Calgary, Alberta, Canada.

Both reduced ANOVAs (model 1: *R^2^*_adj_ = 0.04, F = 2.51, *P = *0.02, *n* = 231; model 2: *R^2^*_adj_ = 0.09, F = 4.33, *P = *0.0004, *n* = 207) retained year-by-zone interactions (model 1: F_2,224_ = 3.34, *P* = 0.04, model 2: F_2,200_ = 3.76, *P* = 0.03). Juveniles born in 2008 tended to be smallest, but year effects (model 1: F_1,224_ = 5.61, *P = *0.02; model 2: F_2,200_ = 9.92, *P = *0.0001) were only significant in the transition zone, and the effect of zone (model 1: F_2,224_ = 0.47, *P = *0.63; model 2: F_1,200_ = 0.02, *P = *0.88) was not significant in any year (Fig. 6A,B). In general, females were larger than males (Fig. 6A,B), significantly so according to model 2 (F_1,200_ = 4.36, *P = *0.04), but not quite according to model 1 (F_1,224_ = 3.64, *P = *0.058).

### Reproductive phenology

There did not seem to be a consistent pattern in terms of among-zone differences in phenology (Table S3). However, compared to the other two years, events occurred later in 2008 and the lactation period seemed to last longer. Fledging was delayed in 2008, when 9% of juveniles were captured in July, compared to roughly half of those born in 2006 and 2007. Furthermore, only in 2008 did we capture newly fledged bats well into September, some of which exhibited much less wing development, e.g., larger epiphyseal gaps indicative of more recent fledging [41], than even the first juveniles captured in the other two years.

In summary, body condition of bats was generally maximized in the transition zone, but not different between the urban and rural zones (although the condition of lactating females was better in the urban than in the rural zone). The summer of 2008 was also when bats in all three zones were generally in the worst condition. Whereas the reproductive rates of adult females did not vary among zones, the reproductive percentage of adult males in 2008 was higher in the urban zone than in the transition zone. Mean caudal swelling (an index of spermatogenesis) was similar among zones. The proportion of juveniles (an index of reproductive success) was generally maximized in the transition zone, and in 2008, it was higher in the urban zone than in the rural zone. Among-year differences in reproductive rates and juvenile production were only observed in the urban zone, where the female reproductive rate was highest in 2006, the male reproductive rate was highest in 2008, and the production of juveniles was highest in 2008. Mean forearm length of juveniles (an index of post-natal growth) was unaffected by zone, but transition-zone juveniles born in 2008 were smaller than those born in 2006 or 2007. Finally, most reproductive events tended to occur later in 2008 than in 2006 or 2007.

## Discussion

There is growing interest in the demographic responses of wild animals to urbanization, although findings from urban autecological studies (mainly avian) are equivocal. For example, nesting in an urban area was not detrimental to the reproductive success of birds in several studies [46], [47], [48], [49], whereas others reported reduced fledging success in relation to urbanization [50], [51]. A more limited literature suggests that the demographic effects of urbanization on mammals are also variable. Urbanization enhanced the reproduction and survival of synanthropes such as fox squirrels *Sciurus niger* Geofroy [52] and raccoons (*Procyon lotor*) [53]. It may also benefit non-synanthropes. Urban black bears (*Ursus americanus*) had greater body mass and productivity than non-urban bears [54], and predation on hedgehogs (*Erinaceus europaeus*) was lower in urban than in rural areas [55]. In contrast, urbanization did not affect the body mass and annual survival of striped skunks (*Mephitis mephitis*) [56], and was detrimental to both the recruitment and survivorship of Key deer (*Odocoileus virginianus clavium*) [57] and the survival of wood mice (Apodemus sylvaticus) [58].

A five-year study of another synanthropic bat, *E. fuscus*, in Fort Collins, Colorado [59] observed female reproductive rates and juvenile survival that were high compared to range-wide values. Urbanization may have favoured the population growth and range expansion of *E. fuscus*, possibly by increasing the availability of buildings [59], in which they roosted exclusively (T. O'Shea, pers. comm.). However, O'Shea et al. [59] did not examine how bat demography differed between urban and non-urban populations. Thus, ours is the first study to compare urban and non-urban bats in such a way that inferences may be drawn about how urbanization affects bat demography, and it assesses a novel hypothesis: that city life offers Prairie bats a fitness advantage. The most direct measures of fitness are lifetime reproductive success [60] and lifetime recruitment [61], which incorporate fecundity and, especially in long-lived species, adult survivorship. However, both parameters are often hard to estimate in natural populations [62]. This is especially true for species such as the little brown bat, which has a maximum longevity>30 years [63], making it necessary to use less direct estimators of fitness.

Body condition was the most fundamental measure of fitness we considered. It reflects individual foraging success, and therefore phenotypic fitness [64], and influences other parameters related to lifetime reproductive success. For bats, it may affect: whether or not they reproduce [18], the reproductive success of males (assuming volume of sperm produced predicts mating success) [22] and females [18], and the likelihood of hibernating successfully [65]. Given the hypothesis that urbanization increases prey availability while decreasing the costs of flight, we expected urban bats to be in better body condition than non-urban conspecifics. Instead, bats consistently did best in the transition zone, where juveniles also gained mass more rapidly compared to their urban counterparts.

Reproductive rates, or the proportions of adults that were reproductively active each year, are fairly mutable population parameters, as might be expected in long-lived animals, and are especially variable in temperate-zone bats, whose decision to reproduce may depend on extrinsic (roost and food availability, weather) and intrinsic (body condition) factors [18]. Given the hypothesis that urbanization enhances all these factors for bats in the Prairies, we expected urban bats to have the highest reproductive rates. Instead, female rates were similar among zones. Male rates were more variable, and differed among zones in 2008, when the transition zone had the fewest reproductive males. These results not only refute our hypothesis, but they also indicate that the consistent differences in adult body condition among zones did not cause reproductive rates to vary. They also did not affect the volume of sperm produced, despite the correlation between individual testicular/caudal swelling and body condition. This suggests the existence of body condition thresholds below which females may not reproduce and spermatogenesis may be compromised, but which were not attained in this study. Also, all studies that have examined variation in the reproductive rates of bats on an annual basis or in relation to habitat quality [18], [35], [59], [66], [67], [68] have considered females. This is not surprising. Females invest more in reproduction than males do during the summer [35], so when conditions are poor, the consequences of raising a pup to independence should theoretically outweigh those of producing sperm. However, these results underscore how poorly we understand the ecology of male bats, whose reproductive rates may actually be more variable than those of females in certain situations. Thus, future studies of the demography of temperate-zone vespertilionid bats should place more emphasis on male reproduction than in the past.

Contrary to the prediction that urban bats would have the greatest reproductive success (as indexed by the ratio of volant juveniles to post-parturient females), transition-zone bats generally did best, a result we hypothesize is directly related to their better body condition. An improvement in maternal body condition should not only increase the energy allocated to milk production, but also increase survivorship [69], both of which should enhance fledging success. Furthermore, mortality of juvenile bats may occur mainly during the post-fledging stage, as a result of poor body condition [70]. The fact that our prediction was not supported by our data could also indicate that our belief that urban bats experience reduced predation risk needs revising given recent findings that tawny owls (*Strix aluco*) were more likely to consume bats in urban than in non-urban areas [71]. We observed great horned owls (*Bubo virginianus*), which prey on bats [72], throughout our study area, but at more rural and urban sites than transition-zone sites. Black-billed magpies (*Pica pica*) were also common in our study area, but nowhere more so than in the city, and they are documented predators of little brown bats [73], as we observed. Interestingly, only in the urban zone did we ever observe emerging little brown bats exhibiting what seemed to be anti-predator behaviour.

We had no *a priori* prediction as to the effect of urbanization on size of volant juveniles, but we did not observe one, perhaps because of an absolute requirement for pups to attain skeletal dimensions close to those of adults before they can fly [74]. However, only in the transition zone were bats born in 2008 smaller than bats born in 2006 or 2007, as might be expected given poorer maternal body condition [69] and inclement weather [45]. Because lift and manoeuvrability are inversely related to wing loading, bats with shorter wings may have increased flight costs and reduced hunting success [75]. Why this year effect was not detected in rural and urban bats is hard to explain, but a 1-mm-difference in mean forearm length may not be biologically significant.

Findings that several aspects of the fitness of little brown bats were maximized in the transition zone were surprising. This zone was an artificial construct, included simply to separate urban and rural bat assemblages, and its size was based on foraging ranges of much larger *Lasiurus cinereus* [31], and yet, it turned out to be biologically relevant for little brown bats. Furthermore, had we hypothesised that urbanization is detrimental to fitness, we would have predicted that rural individuals are in the best condition, as was observed, for example, in house sparrows (*Passer domesticus*) in Hungary [76]. Thus, we made no explicit predictions regarding the relative fitness of transition-zone bats – if anything, we would have expected them to exhibit intermediate, or perhaps the most variable, fitness – and we might have omitted them from the analysis had they not produced such striking results.

Little brown bats in the transition zone had increased fitness even though this was where insect availability was lowest and individual foraging rates did not differ among zones [31]. Thus, this result does not seem to reflect patterns of prey availability or feeding activity. Rather, we hypothesize that among-zone variation in bat fitness reflects among-zone variation in food competition. The difference between competition in the urban and transition zones may partly reflect density-dependent effects because the urban bat assemblage is characterized by a marked increase in the abundance of little brown bats [31]. Adams [77] found that compared to adults, newly fledged little brown bats, being less manoeuvrable, were restricted to hunting in relatively open microhabitats. The reaction of adults to the appearance of these volant juveniles, i.e., potential competitors, depended on population density. When it was high, they moved into more cluttered microhabitats (where the energetic costs of flight were greater) and switched to less profitable prey. When it was low, they did not alter their foraging behaviour. Roost type and colony size may also be relevant. With the exception of one tree colony, urban maternity roosts (n = 6) in our study were in human structures, with colony sizes ranging from 52 to >500 adult females. Similarly, the two rural colonies (both in attics) were large: one had >250 and the other had >1,000 adult females. In contrast, transition-zone bats (n = 6 roosts) roosted in trees or under shingles, and the largest colony (in a tree) had 265 adult females. Larger colonies in artificial than in tree roosts, while not unusual [78], may be detrimental to fitness. Reduced reproductive success as a function of increasing colony size is documented in *Myotis griescens* [79] and *M. myotis* [80]. Increased competition in larger colonies may force bats to spend more time foraging [79] or to forage further from their roosts [81], although the latter may not be an option for bats in the rural zone, where the distribution of suitable habitat was patchiest [31]. Finally, the fact that adult sex-ratios were most even in the transition zone [31] may contribute to reduced food competition because males have lower energy requirements than females do [81]. Thus, the trend toward reduced prey availability in the transition zone may not pose a constraint if bats can meet their energy needs in a shorter period of time or forage closer to their roosts.

That we detected annual variation in reproductive rates and reproductive success only in the urban zone could be an artefact of small sample sizes of rural and transition-zone bats. Nevertheless, absolute differences in proportions of juvenile bats between any two years were greatest in the urban zone. Thus, urban populations may be less buffered than non-urban populations against yearly variation in reproductive success. Similarly, increased inter-annual variation of demographic parameters as a consequence of urbanization have been observed in American crows (*Corvus brachyrhynchos*) [82] and fox squirrels [83]. The fact that male reproductive rates and reproductive success of urban bats were highest in 2008 may be related to weather. By increasing insect abundance, an increase in precipitation may enhance survival of [68] and reproduction by little brown bats, especially if it occurs during the lactation period [84]. Indeed, the amount of rainfall in Calgary during the lactation period was highest in 2008, and insect availability was higher in 2008 than in 2007 [31]. At the same time, wet weather may be associated with reproductive delays and reduced body condition [66], both of which were observed in 2008.

Our data did not support the hypothesis that urbanization benefits little brown bats in grassland ecosystems. Urban bats may be more abundant, but there was no evidence that they were fitter than non-urban conspecifics, underscoring the fact that population density can be a misleading indicator of habitat quality [85]. Ultimately, we reject the idea that the urban area is a population source, and instead postulate that it could be a population sink. Furthermore, relative to the transition zone, the urban zone was characterized by a more female-biased adult sex ratio [31] and an increased male reproductive rate in 2008. Additionally, among-zone variation in body condition was not associated with among-zone variation in reproductive rates. Thus it seems that urban individuals may opt to reproduce to the detriment of their body condition and potentially to their future survival [43]. If little brown bats erroneously perceive a Prairie city as offering higher quality habitat than the surrounding landscape (perhaps due to increased roost availability or habitat connectivity), and are therefore drawn to it, then the city could be an ecological trap [86], [87]. Solid evidence of a habitat sink or ecological trap resulting from urban development is rare [86], and without data on immigration, emigration and mortality, the existence of source-sink dynamics cannot be conclusively established.

Given that little brown bats are highly synanthropic [78] and that increased urban abundance of commensal animals often reflects their enhanced demographic performance [88], we judged this to be an ideal study system to address our hypothesis. In other words, if urbanization benefits any bats anywhere, it seems especially likely to do so for little brown bats in the Prairies, where the availability of roosts and prey may be limited by landscape structure. The inability to detect any such benefits suggests that urbanization may be universally detrimental to the fitness of at least this species and perhaps other bats, and this may be cause for concern under the current scenario of rapid urban expansion.

## Supporting Information

Table S1 **Mass data by age, sex and adult female reproductive status for** ***M. lucifugus*** **captured from 2006 to 2008 in and near Calgary, Alberta, Canada.**
(DOC)

Table S2 **Reproductive phenology of** ***M. lucifugus*** **in the rural, transition and urban zones, from 2006 to 2008, in and near Calgary, Alberta, Canada.**
(DOC)

Table S3 **Numbers of individuals per age, sex and adult reproductive-status category, for** ***M. lucifugus*** **captured from 2006 to 2008 in and near Calgary, Alberta, Canada.** Within-year recaptures are omitted from total numbers of bats in each age/sex category, but not necessarily from numbers in each adult reproductive status category, if reproductive status changed from first to second capture. R = reproductive (undergoing spermatogenesis), NR = non-reproductive, NOP = not obviously pregnant (female captured before date on which we captured the first lactating female in any year, fetus undetected), P = pregnant, L = lactating, PL = post-lactating.
(DOC)
