# Supplementary material for: Influence of Urbanization on Demography of Little Brown Bats (Myotis lucifugus) in the Prairies of North America
Source: PLoS One. 2011 May 25;6(5):e20483. doi: 10.1371/journal.pone.0020483 (PMC3154510; doi:10.1371/journal.pone.0020483)
Supplement: Table S1 — Mass data by age, sex and adult female reproductive status for M. lucifugus captured from 2006 to 2008 in and near Calgary, Alberta, Canada. (DOC) [file pone.0020483.s001.doc]

Table S1. Mass data by age, sex and adult female reproductive status for *M. lucifugus* captured from 2006 to 2008 in and near Calgary, Alberta, Canada.

|  | Adults | | | | | | |  | Juveniles | |
| --- | --- | --- | --- | --- | --- | --- | --- | --- | --- | --- |
|  | Female | | | | |  | Male |  | Female | Male |
|  | L | NR | P | PL | All |  | All |  |  |  |
| Mean ± SE | 8.94 ± 0.07 | 8.36 ± 0.06 | 10.14 ± 0.09 | 9.40 ± 0.18 | 8.75 ± 0.04 |  | 8.11 ± 0.07 |  | 7.40 ± 0.08 | 7.24 ± 0.08 |
| Minimum | 6.9 | 5.9 | 8 | 6.6 | 5.4 |  | 5.2 |  | 5.6 | 5.2 |
| Maximum | 12.3 | 12.9 | 13.4 | 13.5 | 13.5 |  | 13.7 |  | 10.7 | 10.2 |
| n | 200 | 214 | 175 | 59 | 957 |  | 254 |  | 147 | 117 |

Female reproductive conditions: L=lactating, NR=non-reproductive, P=pregnant, PL=post-lactating.
