# Supplementary material for: Influence of Urbanization on Demography of Little Brown Bats (Myotis lucifugus) in the Prairies of North America
Source: PLoS One. 2011 May 25;6(5):e20483. doi: 10.1371/journal.pone.0020483 (PMC3154510; doi:10.1371/journal.pone.0020483)
Supplement: Table S2 — Reproductive phenology of M. lucifugus in the rural, transition and urban zones, from 2006 to 2008, in and near Calgary, Alberta, Canada. (DOC) [file pone.0020483.s002.doc]

Table S2. Reproductive phenology of *M. lucifugus* in the rural, transition and urban zones, from 2006 to 2008, in and near Calgary, Alberta, Canada.

|  | | Pregnancy | | Lactation | | | | Weaning | Fledging | | Spermatogenesis |
| --- | --- | --- | --- | --- | --- | --- | --- | --- | --- | --- | --- |
| Year | Zone | First | Last | First | Last | Median | Interval (d) | First | First | Median | First |
| 2007 | rural | 176 | 230 | 193 | 225 | 193 | 32 | 208 | 193 | 208 | 205 |
| 2008 | rural | 180 | 180 | 180 | 235 | 214 | 55 | 191 | 191 | 235 | 180 |
| 2006 | transition | 173 | 188 | 188 | 215 | 195 | 27 | 215 | 195 | 215 | 169 |
| 2007 | transition | 166 | 166 | 192 | 206 | 195 | 14 | 206 | 202 | 206 | 171 |
| 2008 | transition | 184 | 184 | 193 | 225 | 225 | 32 | 225 | 225 | 246 | 186 |
| 2006 | urban | 153 | 187 | 177 | 214 | 187 | 37 | 214 | 193 | 207 | 177 |
| 2007 | urban | 159 | 188 | 178 | 214 | 194 | 36 | 200 | 191 | 227 | 181 |
| 2008 | urban | 172 | 189 | 189 | 236 | 210 | 47 | 210 | 210 | 236 | 189 |

Numbers are Julian days on which evidence of a given event was observed. Weaning refers to the appearance of post-lactating females in the population and fledging refers to the appearance of volant juveniles.
