# Supplementary material for: Influence of Urbanization on Demography of Little Brown Bats (Myotis lucifugus) in the Prairies of North America
Source: PLoS One. 2011 May 25;6(5):e20483. doi: 10.1371/journal.pone.0020483 (PMC3154510; doi:10.1371/journal.pone.0020483)
Supplement: Table S3 — Numbers of individuals per age, sex and adult reproductive-status category, for M. lucifugus captured from 2006 to 2008 in and near Calgary, Alberta, Canada. Within-year recaptures are omitted from total numbers of bats in each age/sex category, but not necessarily from numbers in each adult reproductive status category, if reproductive status changed from first to second capture. R = reproductive (undergoing spermatogenesis), NR = non-reproductive, NOP = not obviously pregnant (female captured before date on which we captured the first lactating female in any year, fetus undetected), P = pregnant, L = lactating, PL = post-lactating. (DOC) [file pone.0020483.s003.doc]

Table S3. Numbers of individuals per age, sex and adult reproductive-status category, for *M. lucifugus* captured from 2006 to 2008 in and near Calgary, Alberta, Canada. Within-year recaptures are omitted from total numbers of bats in each age/sex category, but not necessarily from numbers in each adult reproductive status category, if reproductive status changed from first to second capture. R=reproductive (undergoing spermatogenesis), NR=non-reproductive, NOP=not obviously pregnant (female captured before date on which we captured the first lactating female in any year, fetus undetected), P=pregnant, L=lactating, PL=post-lactating.

|  |  |  | All adults | |  | Juveniles | |  | Adult males | |  | Adult females | | | | |
| --- | --- | --- | --- | --- | --- | --- | --- | --- | --- | --- | --- | --- | --- | --- | --- | --- |
| Zone | Year |  | Female | Male |  | Female | Male |  | R | NR |  | NOP | P | L | PL | NR |
| Transition | 2006 |  | 61 | 25 |  | 12 | 10 |  | 15 | 8 |  | 15 | 20 | 18 | 3 | 5 |
| Urban | 2006 |  | 165 | 32 |  | 12 | 10 |  | 14 | 18 |  | 45 | 40 | 57 | 3 | 10 |
| Rural | 2007 |  | 94 | 9 |  | 11 | 19 |  | 4 | 5 |  | 54 | 4 | 19 | 2 | 11 |
| Transition | 2007 |  | 49 | 32 |  | 10 | 9 |  | 15 | 16 |  | 22 | 1 | 8 | 3 | 11 |
| Urban | 2007 |  | 184 | 70 |  | 15 | 13 |  | 32 | 38 |  | 72 | 14 | 37 | 7 | 50 |
| Rural | 2008 |  | 128 | 11 |  | 17 | 12 |  | 9 | 2 |  | 51 | 21 | 26 | 12 | 14 |
| Transition | 2008 |  | 73 | 25 |  | 13 | 26 |  | 12 | 12 |  | 47 | 1 | 7 | 10 | 5 |
| Urban | 2008 |  | 278 | 68 |  | 53 | 34 |  | 55 | 12 |  | 96 | 76 | 33 | 21 | 46 |
